# Detecting Disordered Eating Behaviors in Greek Youth with Type 1 Diabetes Mellitus by Using the Diabetes Eating Problem Survey—Revised (DEPS-R): Associations with Insulin Restriction, Glycemic Control, and Anthropometric Parameters

**DOI:** 10.3390/children12060795

**Published:** 2025-06-18

**Authors:** Anastasia Oikonomou, Athanasios Christoforidis, Eleni P. Kotanidou, Ioanna Giannopoulou, Eleni Paschalidou, Vasiliki Rengina Tsinopoulou, Georgia Sotiriou, Kyriaki Tsiroukidou, Assimina Galli-Tsinopoulou

**Affiliations:** 1 Program of Postgraduate Studies “Adolescent Medicine and Adolescent Health Care”, School of Medicine, Faculty of Health Sciences, Aristotle University of Thessaloniki, 54124 Thessaloniki, Greece; 2 1st Department of Pediatrics, School of Medicine, Faculty of Health Sciences, Aristotle University of Thessaloniki, Ippokratio General Hospital, 54642 Thessaloniki, Greece; 3 2nd Department of Pediatrics, School of Medicine, Faculty of Health Sciences, Aristotle University of Thessaloniki, University General Hospital AHEPA, 54636 Thessaloniki, Greece; 4 2nd Department of Psychiatry, Faculty of Medicine, School of Health Sciences, National and Kapodistrian University of Athens, Attikon University General Hospital, 12462 Athens, Greece; 5 3rd Department of Pediatrics, School of Medicine, Faculty of Health Sciences, Aristotle University of Thessaloniki, Ippokratio General Hospital, 54642 Thessaloniki, Greecektsiroukidou@gmail.com (K.T.)

**Keywords:** diabulimia, DEPS-R, adolescents, type 1 diabetes Mellitus, eating disorders, disordered eating behaviors

## Abstract

**Background/Objectives**: This study assesses the prevalence of diabulimia in Greek children and adolescents with Type 1 Diabetes Mellitus (T1DM) by using the Diabetes Eating Problem Survey—Revised (DEPS-R) questionnaire and addresses a gap in the literature on eating disorders (EDs) and disordered eating behaviors (DEBs) in this population. The DEPS-R threshold score of ≥20, although originally established in international studies, has also been applied in Greek adult validation studies. However, it has not yet been formally validated in Greek youth. **Methods**: Participants aged 9–18 years, diagnosed with T1DM a minimum of one year before the start of the study, were recruited from three pediatric departments in Thessaloniki and were asked to complete the Greek version of the DEPS-R questionnaire. Appropriate statistical analysis was employed to investigate the association of the DEPS-R score with anthropometric, demographic, and glycemic variables derived from the clinical assessment and the patient’s medical records. **Results**: Girls had significantly higher DEPS-R scores compared with boys. Significant positive associations were observed between the DEPS-R score and both age (r = 0.212, *p* = 0.020) and Body Mass Index (BMI) (r = 0.419, *p* < 0.001). A significant association with Glycated Hemoglobin (HbA1c) (r = 0.182, *p* = 0.047) suggested that poorer glycemic control may be linked to disordered eating, although no significant associations were identified with physical activity or type of insulin therapy. **Conclusions**: Older age, higher Body Mass Index (BMI) and elevated Glycated Hemoglobin (HbA1c) levels are associated with increased risk of disordered eating in youth with T1DM, especially in girls. Therefore, the implementation of early screening and targeted interventions is imperative.

## 1. Introduction

Type 1 Diabetes Mellitus (T1DM) results from complex interactions among genetic, autoimmune, and environmental factors, leading to the autoimmune destruction of pancreatic beta cells, which substantially reduces or eliminates insulin production [1]. Autoimmune T1DM is the most common metabolic disease in pediatric patients [2]. The incidence rate in childhood has increased considerably worldwide by 3–4% compared with the early 1990s [2,3]. The diagnostic time for T1D may vary, as it can be detected at any age during childhood, with the risk of developing stage 2 (islet autoimmunity and dysglycemia) or stage 3 (hyperglycemia meeting glycemic and clinical diagnostic criteria) being proportionally associated with aging in patients presenting islet autoantibodies positivity, especially after the period of early childhood [4]. Environmental factors such as viral infections, seasonal changes, dietary habits, and vitamin D levels may trigger T1DM [4,5,6].

The management of T1DM in children and adolescents requires a comprehensive approach involving regular glucose monitoring, insulin therapy, and nutritional guidance. The latter includes adherence to a Mediterranean style dietary plan, which is mostly followed in Mediterranean countries [7,8,9], although nutritional recommendations may vary depending on local clinical practices and cultural influences. Adherence to the International Society for Pediatric and Adolescent Diabetes (ISPAD) guidelines is crucial to avoiding complications, and a multidisciplinary team is essential to effective care [10]. The management of T1DM during adolescence is pivotal, as this portion of patients are at increased risk of developing disordered eating behaviors (DEBs), which is mainly attributed to their increased psychosocial vulnerability [10,11]. Several long-term transition studies have demonstrated the crucial role of structured and multidisciplinary care that focuses on metabolic and psychological improvement [11,12]. Adolescence brings significant physical, hormonal, and psycho-emotional changes, complicating disease management [13]. Adolescence-related factors, both non-modifiable (pubertal hormonal changes) and modifiable (behavioral pattern changes), may lead to the deterioration of glycemic control and increase the risk of developing complications that are both acute (such as diabetic ketoacidosis or hypoglycemia) and long-term (diabetic retinopathy, nephropathy, etc.) [14,15,16,17,18].

EDs and disordered eating behaviors (DEBs) are prevalent among children and adolescents with T1DM, with significantly increased prevalence compared with individuals without a diagnosis of diabetes [19,20,21]. DEBs such as food restriction and insulin omission are particularly common, affecting 30–50% of girls and 10–20% of boys with T1DM [22]. Diabulimia is defined as the deliberate omission or underuse of insulin in individuals with T1DM aiming to control their body weight [23]. Although not officially recognized as a distinct diagnostic category in DSM-5, diabulimia represents a high-risk behavior pattern, which significantly increases the risk of acute and long-term diabetes complications, including diabetic ketoacidosis, retinopathy, neuropathy, and premature mortality [22,24]. Risk factors for EDs and DEBs include age, biological sex, body dissatisfaction, and family environment [25].

The early detection of DEBs by using appropriate tools such as the Diabetes Eating Problem Survey—Revised (DEPS-R) is crucial to early intervention and the improvement in psychosocial outcomes in this population [1,19,26,27,28,29,30,31]. A DEPS-R total score of ≥20 has been suggested as a clinically relevant threshold for detecting individuals with T1DM who are at increased risk of DEBs, including poor glycemic control, diabulimia, and body image concerns [29]. This cut-off has an acceptable sensitivity and specificity in adolescent populations and has also been applied in a Greek adult validation study [30,32,33,34]. Meanwhile, this cut-off is considered clinically relevant, as it is associated with higher rates of diabetes-related complications and suboptimal disease management [23,29]. Insulin restriction as a weight control strategy is in the spotlight for the management of disordered eating patterns in T1DM patients, whereas insulin mismanagement should be considered within the broader context of EDs. Additionally, it has to be underlined that the so-called "diabulimia", constitutes a term that significantly lacks formal diagnostic recognition and may not adequately encompass the full spectrum of these behaviors.

Given the complexity of these behaviors, this study is the first to focus on Greek children and adolescents with T1DM, addressing a significant gap in the literature. While cultural and social norms surrounding food, body image, and health behaviors may significantly influence the prevalence and manifestation of EDs, further research is required to explore their specific impact in the Greek context. The deeper insight into the demographic, anthropometric, and glycemic factors that our findings provide may contribute to a more comprehensive understanding of the multifactorial nature of DEBs in youth with T1DM. The objectives of this study were to assess the risk of DEBs in Greek youth with T1DM by using the DEPS-R questionnaire and examine associations with demographic, anthropometric, and glycemic control parameters.

## 2. Materials and Methods

### 2.1. Study Population

This study was designed as a cross-sectional observational study aimed to assess the risk of DEBs in children and adolescents with T1DM by using the DEPS-R questionnaire. It focused on the application of the Greek version of the DEPS-R questionnaire in pediatric patients aged 9–18 years. Data collection was conducted anonymously and voluntarily between December 2023 and May 2024 in a sample of 120 Greek patients (61 boys and 59 girls) diagnosed with T1DM at least 1 year before the start of the study. All participants were proficient in reading and writing Greek and were capable of understanding and completing the questionnaires. The DEPS-R questionnaire was administered in person by trained personnel (a pediatric diabetes nurse and a postgraduate medical researcher), following standardized procedures to ensure consistency, confidentiality, and non-influence. The administration took place before the routine consultation with the pediatric diabetologist, in a quiet consultation room within the outpatient clinic. Participants were encouraged to complete the questionnaire independently and in a calm environment without time constraints. The parents’ or guardians’ presence depended on the child’s request. This setting aimed to minimize external influences and ensure the validity of the self-reported responses.

**Figure 1:**
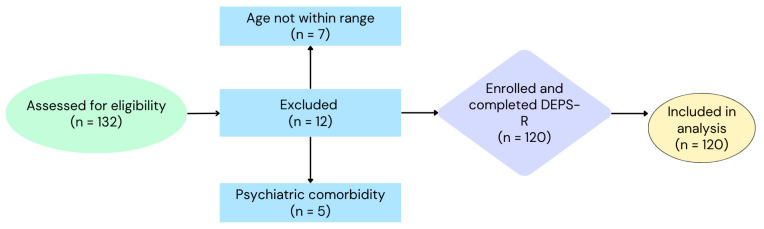
Recruitment flow of participants from initial screening to final analysis.

The study sample was recruited from the Pediatric Diabetes Outpatient Clinics of the 2nd Department of Pediatrics of Aristotle University of Thessaloniki at AHEPA University General Hospital, the 1st Department of Pediatrics of Aristotle University of Thessaloniki at Ιppokratio Hospital, and the 3rd Department of Pediatrics of Aristotle University of Thessaloniki at Ιppokratio Hospital. A total of 132 patients were assessed for eligibility during routine visits to the participating pediatric diabetes clinics. Twelve individuals were excluded due to not meeting the age criteria (*n* = 7) or having psychiatric comorbidities requiring psychotropic medication (*n* = 5). The remaining 120 eligible participants (61 boys and 59 girls) were enrolled in this study, completed the DEPS-R questionnaire, and were included in the final analysis. The recruitment process is summarized in Figure 1. Participants provided assent, and their parents or guardians gave informed consent prior to their inclusion in this study. The exclusion criteria included psychiatric disorders requiring psychotropic medication and ages outside the 9–18-year range.

### 2.2. Study Tools

The DEPS-R is a validated screening tool for identifying the risk of DEBs specifically in patients with T1DM and was used to assess disordered eating tendencies and explore their relationship with demographic, anthropometric, and glycemic parameters [29]. Originally, the Diabetes Eating Problem Survey (DEPS) consisted of 28 items. In 2010, Markowitz et al. revised and shortened it to a 16-item questionnaire, enhancing its applicability in both clinical and research settings [29]. The DEPS-R utilizes a 6-point Likert scale for each item, ranging from 0 ("never") to 5 ("always"), with higher scores indicating greater concerns related to eating behaviors and diabetes management. The total score ranges from 0 to 80, calculated by summing the responses of all items. A score above 20 suggests an increased risk of DEBs and may warrant further evaluation or referral to a mental health professional for the clinical assessment and diagnosis of a potential ED, in line with current practice guidelines [29]. A total score of ≥20 on the DEPS-R was used in this study as the threshold indicative of increased risk of DEBs based on previously published validation studies and its consistent international utilization in the adolescent population [30,32,33,34].

The Greek version of the DEPS-R, validated in previous studies, was adapted to improve clarity for a younger population. The internal consistency of the questionnaire has been reported as high, with a Cronbach’s alpha of 0.89 overall (0.90 in females and 0.85 in males), indicating strong reliability [30]. Participants completed the DEPS-R, which comprises 16 questions for the assessment of behaviors related to eating, body image, insulin use, and diabetes self-management [1,19,26,27,28,29,30,31]. Anthropometric data, such as height, weight, Body Mass Index (BMI), and waist circumference, as well as blood pressure, frequency of blood glucose measurements, HbA1c levels, daily meals, and physical activity, were recorded. The waist-to-height index (WHtI) was also recorded as waist circumference divided by height, both in centimeters. Absolute values of anthropometric parameters were converted into standard deviation scores (Z-scores) based on CDC (Centers for Disease Control and Prevention) reference values [35]. The type of insulin therapy (multiple daily injections or insulin pump) was documented. Physical activity was also assessed via a self-reported, single-item question regarding the frequency of exercise in a typical week. Participants were asked to choose among three predefined options: "Not at all" (0 times/week), "Rarely" (1–2 times/week), and "Frequently" (3 or more times/week).

### 2.3. Ethical Considerations

This study was conducted in accordance with the Declaration of Helsinki. The study protocol, consent forms, and the DEPS-R questionnaire were reviewed and approved by the Scientific Council of AHEPA University General Hospital and the Department of Quality Control, Research and Continuing Education of Ιppokratio Hospital (IRB numbers: 10025/27 February 2024 and 12392/21 March 2024, respectively).

### 2.4. Statistical Analysis

The study data were initially recorded using a spreadsheet program and later analyzed using statistical software. Questionnaires were completed during the patients’ routine outpatient visits, after their scheduled medical examination. In cases where responses were missing, participants were asked to complete any unanswered items before submission. As a result, no missing data were recorded for the variables included in the analysis. Microsoft Excel for Mac (version 16.85) and IBM SPSS Statistics for Mac (version 29.0.1.0) were used for both statistical analysis and graphical representation of the results. The Shapiro–Wilk test was used for datasets with fewer than 50 observations, while the Kolmogorov–Smirnov test was used for datasets with 50 or more observations to evaluate whether the data followed a normal distribution. For parameters following a normal distribution, Student′s *t*-test was used to compare the means of two independent groups, while the ANOVA test was used for comparisons across more than two independent groups. In cases of skewed distribution, the Mann–Whitney U test was used to compare two independent groups, while the Kruskal–Wallis test was used for comparisons across more than two independent groups. The chi-square test was used to compare proportions, including the distribution of participants with DEPS-R scores ≥20 and <20 by sex. Linear correlations were assessed using the Pearson correlation coefficient for normally distributed data and Spearman′s rank correlation coefficient for skewed data. No correction for multiple comparisons was applied. The threshold for statistical significance was set to 0.05 (*p* < 0.05).

## 3. Results

A total of 120 questionnaires were completed (61 by boys and 59 by girls). The demographic and anthropometric data of the patients as well as data from their glycemic control categorized by biological sex and in total are shown in Table 1. Differences between boys and girls were examined using independent samples *t*-tests. No significant sex differences were observed in age (*p* = 0.704), disease duration (*p* = 0.277), weight (*p* = 0.689), BMI (*p* = 0.144), BMI Z-score (*p* = 0.181), HbA1c (*p* = 0.226), or WHtI (*p* = 0.412). However, boys were significantly taller than girls (*p* = 0.016). This sex difference in absolute values of height was diminished when Z-scores were calculated and compared.

Of those who completed the questionnaire for this study, 86 (71.7%) were on multiple daily insulin injections, while 34 (28.3%) were on continuous subcutaneous insulin infusion (pump therapy). The demographic, anthropometric, and glycemic control parameters were categorized based on the treatment method, and no statistically significant differences were observed between the groups.

Regarding physical activity, 17 patients (14.17%) reported not exercising at all, 39 (32.50%) reported exercising rarely, and 64 (53.33%) reported exercising frequently. No statistically significant differences were observed in demographic, anthropometric, or glycemic control parameters based on physical activity levels. Similarly, no significant difference in DEPS-R total scores was found among the three activity groups (*p* = 0.197).

The mean waist circumference was 76.27 ± 12.32 cm (boys: 77.05 ± 12.68 cm vs. girls: 75.47 ± 11.93 cm, *p* = 0.484). The mean systolic blood pressure was 117.26 ± 10.47 mmHg, and the diastolic pressure was 71.17 ± 7.47 mmHg. In boys, systolic and diastolic blood pressure values were recorded as 119.41 ± 10.53 mmHg and 71.89 ± 7.64 mmHg, respectively. Among girls, systolic and diastolic blood pressure values were recorded as 115.03 ± 10.29 mmHg and 70.42 ± 7.27 mmHg, respectively. Participants reported an average of 4.36 ± 0.98 meals per day (boys: 4.48 ± 0.99 vs. girls: 4.24 ± 0.95, *p* = 0.327). The mean frequency of physical activity, rated on a three-point Likert scale (not at all, rarely, or frequently), was self-reported as not at all by 14.2%, rarely by 32.5%, and frequently by 53.3% in the total sample, without significant differences in the distribution of answers per sex group (boys: 11.5% not at all, 29.5% rarely, and 59% frequently vs. girls: 16.9% not at all, 35.6% rarely and 47.5% frequently, *p* = 0.422).

### DEPS-R Scores

The mean DEPS-R total score was 13.89 ± 8.62 in boys and 20.56 ± 12.34 in girls. This difference was statistically significant (*p* = 0.003), with girls exhibiting a 6.67-point higher score (95% CI: 2.85–10.49) compared with boys. The overall mean score in the total sample was 17.17 ± 11.09.

As shown in Table 2, girls were significantly more likely to score ≥20 on the DEPS-R compared with boys, indicating a higher risk of DEBs. The difference was statistically significant (χ^2^(1) = 12.81, *p* < 0.001), confirming the increased prevalence of DEBs among female participants. Additionally, as shown in Table A1 (Appendix A), they reported significantly greater endorsement of specific items related to weight control and body dissatisfaction, including “Losing weight is an important goal to me” (*p* < 0.001), “I feel that it’s difficult to lose weight and control my diabetes at the same time” (*p* < 0.001), “I make myself vomit” (*p* = 0.036), “I feel fat when I take all of my insulin” (*p* = 0.022), “Other people tell me to take better care of my diabetes” (*p* = 0.041), and “I would rather be thin than to have good control of my diabetes” (*p* = 0.042).

The linear correlations between the study parameters and the total DEPS-R score are documented in Table 3.

**Figure 2:**
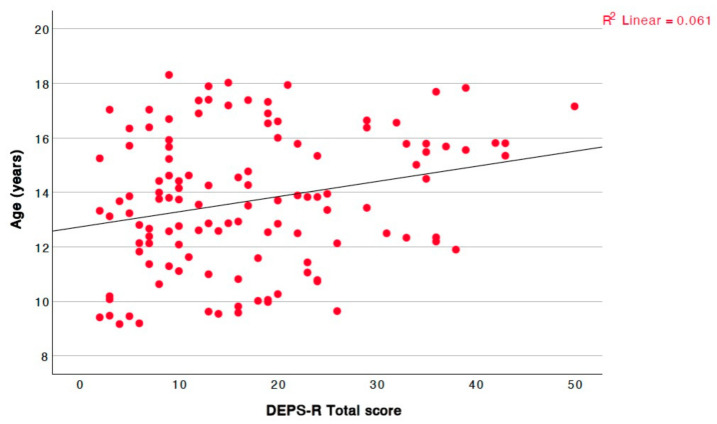
Significant positive linear correlation between total questionnaire score and age (r = 0.212, *p* = 0.020).

The total questionnaire score exhibited a positive linear correlation with age (Figure 2). 

**Figure 3:**
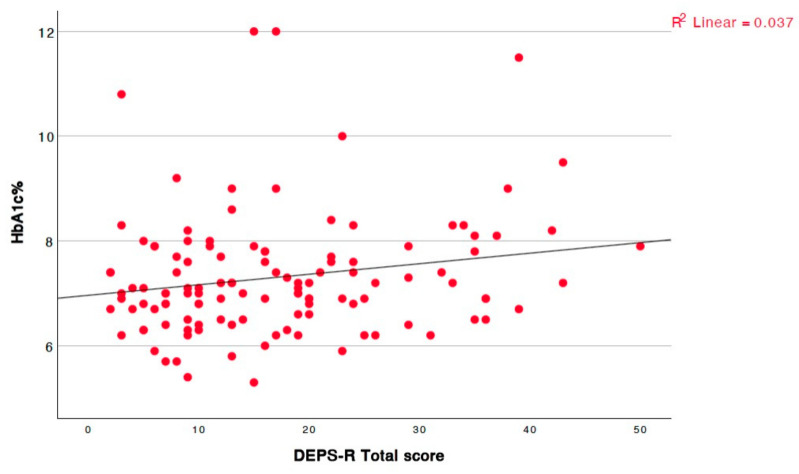
Significant positive linear correlation between total questionnaire score and recent HbA1c (r = 0.182, *p* = 0.047).

A significant positive correlation was also observed between the WHtI and the total DEPS-R score (r = 0.366, *p* < 0.001). To further explore potential gender-specific patterns, correlation analyses were stratified by sex. Among girls, the WHtI showed a strong positive association with the DEPS-R scores (r = 0.607, *p* < 0.001). In contrast, no significant correlations between the DEPS-R and the WHtI (r = 0.100, *p* = 0.443) was observed among boys. Of particular interest, a marginally significant positive correlation was identified between the HbA1c levels and the total questionnaire score (Figure 3). Poorer glycemic control was associated with higher questionnaire scores.

**Figure 4:**
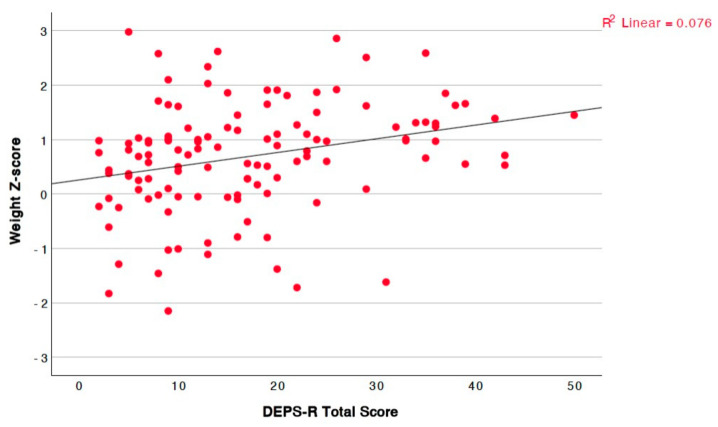
Significant positive linear correlation between total questionnaire score and weight Z-score (r = 0.329, *p* = <0.001).

**Figure 5:**
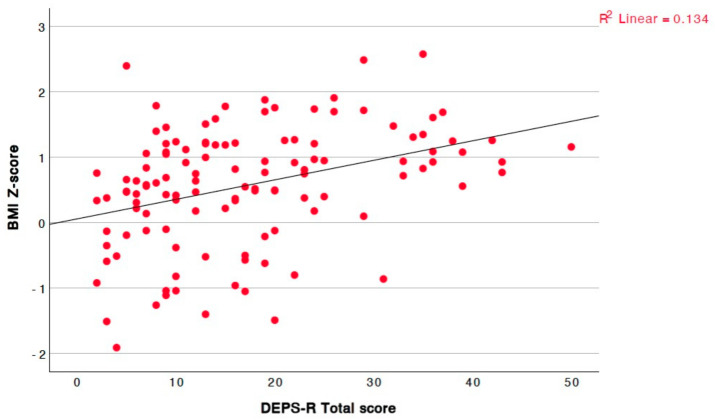
Significant positive linear correlation between total questionnaire score and BMI Z-score (r = 0.394, *p* = <0.001).

Lastly, the score showed a statistically significant positive linear correlation with both the absolute values of weight and BMI, as well as their respective Z-scores (Figure 4 for weight Z-score, Figure 5 for BMI Z-score). In contrast, no linear correlation was observed with height, either in absolute values or Z-scores.

## 4. Discussion

This study investigated DEBs in Greek children and adolescents with T1DM by analyzing DEPS-R scores and their associations with key risk factors. The DEPS-R is a validated screening tool for identifying individuals at risk of DEBs which is utilized for the assessment of disordered eating behaviors and the exploration of their interrelation with demographic, anthropometric, and glycemic parameters. The presence of EDs in individuals with T1DM has been associated with an increased risk of both acute and long-term complications. Acute complications include diabetic ketoacidosis and hypoglycemia, while long-term complications include retinopathy, nephropathy, and neuropathy. In addition, poor glycemic control resulting from DEBs may enhance the risk for macrovascular complications, including cardiovascular disease, cerebrovascular disease, and peripheral arterial disease [12]. This mutually reinforcing relationship between EDs and glycemic control emphasizes the vital importance of early detection and intervention, particularly given that EDs may exacerbate glycemic variability, while poor glycemic control may intensify psychological distress and unhealthy eating patterns [10]. In this context, our study provides insights into the risk patterns observed in the Greek pediatric T1DM population. Girls, older adolescents, and individuals with higher BMI or HbA1c levels were more likely to present with elevated DEPS-R scores, suggesting heightened vulnerability to DEBs. Meanwhile, there is an emerging correlation between overweight and obesity in adolescents and executive functioning impairment, including inhibitory control, which may further compromise their ability to regulate dietary behaviors and diabetes management [36]. Although overweight and obesity were more prevalent among boys (83.6%) than girls (72.9%), only female adolescents exhibited a significant positive association between increased adiposity (BMI and WHtI) and DEB scores, suggesting that alterations in executive functioning may interact with psychosocial factors to increase DEB vulnerability in girls. Given the known psychosocial burden of disordered eating, these results highlight the importance of considering psychosocial dimensions in diabetes care, particularly during adolescence, a developmental stage in which certain individuals may experience increased vulnerability to body image concerns and social pressures [29].

Our study revealed that girls scored significantly higher on the DEPS-R than boys. This finding is consistent with previous studies suggesting that girls are at higher risk of developing DEBs, potentially due to increased body image concerns and social pressures. Furthermore, girls demonstrated elevated scores on specific DEPS-R items related to body dissatisfaction and problematic eating behaviors, thus reinforcing the need for gender-sensitive intervention strategies. Age also emerged as a critical factor, with older adolescents reporting higher total scores on the questionnaire. Such an observation is in agreement with the existing literature, which highlights that the transition to adolescence introduces greater psychosocial challenges, including peer influence and increased sensitivity to body image [10]. This finding suggests that implementing early detection and intervention strategies in younger patients may have the potential to prevent the progression of DEBs as these patients age.

Based on our findings, a significant positive association was identified between DEPS-R scores and BMI, as well as BMI Z-scores, in children and adolescents with T1DM. More particularly, individuals with higher BMI exhibited an increased DEPS-R score, implying a possible association between high BMI and DEBs in youth with T1DM. In addition to BMI, the WHtI was also examined in relation to DEBs. A significant positive correlation was observed in the overall sample, especially among girls. The WHtI may offer complementary insights into the role of body composition in DEBs, particularly in female adolescents with T1DM. These results align with previous studies highlighting body dissatisfaction as a major contributor to disordered eating in youth with T1DM [24]. It is of the utmost importance to address body image concerns as part of routine care for individuals with T1DM, as these issues are closely linked to behaviors that can potentially have a negative impact on glycemic control and long-term diabetes management. However, it is important to note that while these associations were statistically significant, the strength of the correlations, as reflected by the r-values, was modest. This suggests that although variables such as BMI and HbA1c may contribute to the risk of DEBs, they are likely part of a broader, multifactorial framework. The relatively low correlation coefficients highlight the importance of exploring additional psychological, behavioral, and environmental factors that may better account for the observed variance in DEPS-R scores.

Interestingly, our study did not find a significant association between DEPS-R scores and height, whether measured in absolute terms or Z-scores. This result diverges from existing research on physical growth and psychosocial health in youth with T1DM, suggesting that height may play a minimal role in the development of DEBs in the present population [30]. The relationship between HbA1c levels and DEPS-R scores warrants further investigation, as existing findings show only a marginally significant positive association in our study. It should be noted that the mean HbA1c in our sample was relatively close to target levels (7.31 ± 1.16%), which may have limited the variability required to detect stronger associations. Including participants with a broader glycemic range in future studies may help clarify the relationship between glycemic control and DEBs. While our results suggest that poorer glycemic control may be linked to DEBs, including insulin omission as a maladaptive weight control strategy, previous research has shown varied outcomes [19,21]. For example, Peveler et al. (2005) found a strong association between poor glycemic control and DEBs, particularly insulin omission, emphasizing its role in weight management strategies among individuals with T1DM [27]. Similarly, Hevelke et al. (2016) highlighted the prevalence of DEBs in young people with T1DM but noted variability in the strength of its association with HbA1c, influenced by factors such as age and biological sex [26].

Understanding the role of glycemic control in the development of EDs and DEBs is essential to identifying at-risk individuals and implementing tailored interventions in young people with T1DM, with this relationship being bidirectional. On one hand, DEBs can lead to poor glycemic control and elevated HbA1c levels. On the other hand, persistent hyperglycemia and struggle for the achievement of glycemic targets may increase diabetes-related distress, body dissatisfaction, and emotional burden, which can then trigger or worsen DEBs. This cycle is considered pivotal in clinical practice, as it highlights the need for integrated care strategies that address both metabolic control and emotional well-being.

On top of that, these findings highlight the potential value of integrating brief screening tools, such as the DEPS-R, into daily practice in pediatric diabetes clinics. The implementation of such questionnaires may facilitate the early detection of DEBs, particularly in adolescent girls or those with poor metabolic control, and promote a more holistic, multidisciplinary management approach.

The lack of a significant correlation between DEPS-R scores and physical activity levels was an unexpected finding. Previous studies suggest that regular physical activity may protect against EDs and DEBs by enhancing mental well-being and body satisfaction. Nevertheless, our findings suggest that physical activity may not have a substantial influence on eating behaviors in youth with T1DM, potentially due to variations in the type, intensity, or adherence to exercise [29]. This lack of association may be also attributed to the limitations of our assessment method, as physical activity was recorded based on self-reported frequency without accounting for duration, intensity, or motivation. Additionally, psychosocial factors such as fear of hypoglycemia, family support, or body image concerns may mediate the relationship between exercise and DEBs and were not captured in the present study.

Similarly, no significant association was found between insulin regimen and DEPS-R scores, which may reflect the need for more nuanced behavioral data. Future research should consider integrating more detailed behavioral profiling tools that could potentially assess factors such as mealtime patterns, insulin adjustment behaviors, and perceived treatment burden for a better understanding of DEB complexity in this population.

Psychosocial and family-related factors may be crucial mediators in the development of DEBs in youth with T1DM. Although not assessed in the current study, variables such as family conflict, emotional support, depression, anxiety, and diabetes-specific distress have been previously associated with unhealthy eating patterns in adolescents [19,26,31]. For instance, Hevelke et al. highlighted the role of psychosocial burden in shaping eating behaviors in pediatric patients with T1DM [26]. The inclusion of structured psychosocial assessments in future research could significantly improve risk stratification, early support provision, and targeted interventions [29,31]. The integration of validated measures for psychosocial comorbidities, such as anxiety and family functioning, would allow for more comprehensive DEB risk stratification and may reveal mediating pathways not captured by demographic or metabolic data alone.

Based on our findings and the pre-existing literature, we propose a conceptual model in which disordered eating behaviors in adolescents with T1DM result from the interplay of biological, psychological, and behavioral factors. Biologically, higher BMI, older age, and elevated HbA1c levels appear to increase vulnerability to DEBs. Psychologically, factors include body dissatisfaction, low self-esteem, and emotional distress—potentially influenced by social and cultural pressure, which may enhance this risk. Additionally, behavioral and maladaptive practices such as insulin omission or irregular meal patterns may result from internal and external stressors. This multifactorial model underlines the need for integrated care approaches that combine medical, nutritional, and psychological support to address the complex needs of this population [19,26,27,29,31].

We recognize several limitations in our study, such as its cross-sectional nature, which may limit the ability to establish causality or determine DEB development in relation to glycemic control. Furthermore, the absence of a healthy control group limits the ability to make comparisons of the prevalence DEB severity with peers without the diagnosis of T1DM, which would provide valuable context. The inclusion of a comparison group of healthy controls would allow for a more accurate estimation of the excess risk and unique characteristics of DEBs in this clinical population. Additionally, this study does not fully explore the psychosocial and environmental factors that influence DEBs. Another limitation is that the sample was not randomly selected but drawn from a specific clinical population, which may affect the generalizability of the findings to the broader population of children and adolescents with T1DM. Despite these limitations, this study provides valuable preliminary data on DEBs in young patients with T1DM. In order to overcome these limitations, future research should adopt longitudinal designs to examine the temporal dynamics and progression of DEBs, as well as their bidirectional relationships with glycemic control and other influencing factors in this population. Such designs would allow researchers to identify early predictors of DEB onset and track how these behaviors evolve over time in response to developmental, metabolic, and psychosocial changes. To enhance the generalizability of findings and facilitate more meaningful comparisons, it would be beneficial to expand the study population and perform larger and more diverse cohorts, as well as incorporating healthy controls. Furthermore, additional research is required to investigate the impact of psychosocial factors, including anxiety, depression and family dynamics, on eating behaviors and diabetes management, as these factors have been demonstrated to interact with these processes [31]. Last but not least, exploration of the implication of cultural norms and social pressures on body image could also offer a more nuanced understanding of the complex interplay between EDs and DEBs and other factors in different populations.

## 5. Conclusions

This study identifies significant correlations between DEBs and key demographic, anthropometric, and glycemic parameters in children and adolescents with T1DM. Female sex, older age, higher BMI, and elevated HbA1c were significantly associated with higher DEPS-R scores. Our findings highlight associations between DEBs and key clinical and demographic factors, underlining the importance of early detection in routine diabetes care. However, these results should be interpreted with caution due to the cross-sectional design of this study, as it cannot imply causality. However, they highlight the potential value of incorporating DEB screening tools into routine pediatric diabetes care. Future longitudinal studies are needed for the elucidation of the causality and the development of culturally tailored prevention strategies for at-risk individuals.

## Figures and Tables

**Table 1:** Demographic, anthropometric, and glycemic control parameters categorized by sex and in total.

Variable	Boys	CI 95%	Std Error	Girls	CI 95%	Std Error	*p*	Total	CI 95%	Std Error
Age (years)	13.77 ± 2.45	13.14–14.40	0.31	13.60 ± 2.54	12.93–14.26	0.33	0.704	13.69 ± 2.50	13.24–14.14	0.23
Disease duration (years)	5.00 ± 3.24	4.16–5.82	0.42	5.89 ± 3.77	4.91–6.88	0.49	0.277	5.43 ± 3.53	4.80–6.07	0.32
Weight (kg)	58.04 ± 18.13	53.40–62.69	2.32	56.72 ± 17.92	52.05–61.39	2.33	0.689	57.39 ± 17.96	54.15–60.64	1.64
Weight Z-score	0.62 ± 1.06	0.35–0.90	0.14	0.76 ± 0.97	0.51–1.01	0.13	0.469	0.69 ± 1.02	0.51–0.87	0.09
Height (cm)	164.00 ± 15.17	160.00–167.77	1.94	157.86 ± 11.43	154.88–160.84	1.49	0.016	160.92 ± 13.74	158.44–163.40	1.26
Height Z-score	0.59 ± 1.14	0.30–0.88	0.15	0.49 ± 1.13	0.20–0.79	0.15	0.642	0.54 ± 1.13	0.34–0.75	0.10
BMI (kg/m^2^)	21.12 ± 3.67	20.17–22.06	0.47	22.28 ± 4.95	21.00–23.57	0.64	0.144	21.69 ± 4.37	20.90–22.48	0.40
BMI Z-score	0.47 ± 0.89	0.24–0.70	0.11	0.68 ± 0.92	0.44–0.92	0.12	0.181	0.57 ± 0.91	0.41–0.74	0.08
HbA1c (%)	7.21 ± 1.19	6.91–7.52	0.15	7.41 ± 1.13	7.11–7.70	0.15	0.226	7.31 ± 1.16	7.10–7.52	0.11
WHtI (cm)	0.47 ± 0.06	0.45–0.48	0.01	0.48 ± 0.06	0.46–0.49	0.01	0.600	0.47 ± 0.06	0.46–0.48	0.01

BMI: Body Mass Index; HbA1c: Glycated Hemoglobin; WHtI: waist-to-height index. SD: standard deviation. Data are presented as means ± standard deviation (SD).

**Table 2:** Distribution of DEPS-R scores (≥20 vs. <20) by sex.

Sex	DEPS-R < 20 n (%)	DEPS-R ≥ 20 n (%)	Total n (%)	*p*-Value
Males	49 (80.3%)	12 (19.7%)	61 (50.8%)	<0.001
Females	29 (49.2%)	30 (50.8%)	59 (49.2%)	
Total	78 (65.0%)	42 (35.0%)	120 (100.0%)	

DEPS-R: Diabetes Eating Problem Survey—Revised. Data are presented as n (%). *p*-Value for chi-square test for sex distribution across DEPS-R scoring groups.

**Table 3:** Linear correlations of total questionnaire score with study parameters.

Parameter	Correlation with Total Questionnaire Score
Age	r = 0.212, *p* = 0.020, 95% CI = [0.03, 0.38]
Disease duration	r = 0.058, *p* = 0.531
Weight	r = 0.319, *p* = <0.001, 95% CI = [0.15, 0.47]
Weight Z-score	r = 0.329, *p* = <0.001, 95% CI = [0.16, 0.48]
Height	r = 0.104, *p* = 0.260
Height Z-score	r = −0.002, *p* = 0.979
BMI	r = 0.419, *p* = <0.001, 95% CI = [0.26, 0.56]
BMI Z-score	r = 0.394, *p* = <0.001, 95% CI = [0.23, 0.54]
HbA1c	r = 0.182, *p* = 0.047, 95% CI = [0.0, 0.35]

BMI: Body Mass Index; HbA1c: Glycated Hemoglobin. Correlation coefficients (r) were calculated using Spearman’s rho correlation test.

## Data Availability

The original contributions presented in this study are included in the article.

## Abbreviations

The following abbreviations are used in this manuscript:

BMI	Body Mass Index
CDC	Centers for Disease Control and Prevention
DEPS-R	Diabetes Eating Problem Survey—Revised
DEB	disordered eating behavior
ED	eating disorder
HbA1c	Glycated Hemoglobin
PCOS	Polycystic Ovary Syndrome
T1DM	Type 1 Diabetes Mellitus

## Appendix A

**Table A1:** Total questionnaire scores and item-specific scores, with comparisons between boys and girls.

	Boys	Girls	*p*	Total
Total Score	13.89 ± 8.62	20.56 ± 12.34	0.003	17.17 ± 11.09
Losing weight is an important goal to me	1.62 ± 1.60	2.61 ± 1.66	<0.001	2.11 ± 1.70
I skip main meals and/or snacks	1.39 ± 1.14	1.80 ± 1.44	0.161	1.59 ± 1.31
Other people have told me that my eating is out of control	1.03 ± 1.33	1.42 ± 1.56	0.229	1.23 ± 1.45
When I overeat, I don’t take enough insulin	1.01 ± 1.18	1.22 ± 1.20	0.260	1.12 ± 1.19
I eat more when I am alone than when I am with others	1.23 ± 1.40	1.67 ± 1.46	0.071	1.45 ± 1.44
I feel that it’s difficult to lose weight and control my diabetes at the same time	0.72 ± 1.10	1.93 ± 1.80	<0.001	1.32 ± 1.60
Ι avoid checking my blood sugar when I feel it is out of range	1.11 ± 1.34	0.98 ± 1.11	0.825	1.05 ± 1.23
I make myself vomit	0.07 ± 0.25	0.34 ± 0.82	0.036	0.20 ± 0.62
I try to keep my blood sugar high so that I will lose weight	0.46 ± 1.29	0.68 ± 1.23	0.126	0.57 ± 1.28
I eat in a way to get ketones	0.20 ± 0.65	0.17 ± 0.50	0.604	0.18 ± 0.58
I feel fat when I take all of my insulin	0.18 ± 0.62	0.54 ± 1.04	0.022	0.36 ± 0.87
Other people tell me to take better care of my diabetes	1.41 ± 1.53	1.98 ± 1.61	0.041	1.69 ± 1.59
After I overeat, I skip my next insulin dose.	0.61 ± 1.05	0.66 ± 0.94	0.451	0.63 ± 1.00
I feel that my eating is out of control	1.18 ± 1.19	1.61 ± 1.41	0.103	1.39 ± 1.32
I alternate between eating very little and eating huge amounts	1.13 ± 1.13	1.71 ± 1.53	0.060	1.42 ± 1.37
I would rather be thin than to have good control of my diabetes	0.52 ± 1.21	1.22 ± 1.78	0.042	0.87 ± 1.55

SD: standard deviation. Data are presented as means ± standard deviation (SD).
